# A Rare Case of Sequential Contralateral Tubal Pregnancies

**DOI:** 10.7759/cureus.104678

**Published:** 2026-03-04

**Authors:** Elena R Gaston, Shawnequa Brown

**Affiliations:** 1 Obstetrics and Gynecology, Edward Via College of Osteopathic Medicine, Spartanburg, USA; 2 Obstetrics and Gynecology, Medical University of South Carolina Health - Orangeburg, Orangeburg, USA

**Keywords:** bilateral tubal pregnancies, bilateral tubal pregnancy, ectopic pregnancy, ectopic pregnancy treatment, extrauterine pregnancy, laparoscopic management, methotrexate

## Abstract

Bilateral tubal ectopic pregnancies are the rarest form of extrauterine pregnancy, with an incidence higher in women who are undergoing ovulation induction or assisted reproductive techniques. With an unpredictable clinical course and similar presentation to unilateral ectopic pregnancies, it is important to explore the possibility of bilateral tubal pregnancies with known risk factors. Early recognition is essential to prevent morbidity. We report a case of a 26-year-old gravida 3 para 2 female patient who presented to the emergency department with right lower pelvic pain and vaginal bleeding several weeks after a routine Papanicolaou (Pap) smear. The patient is sexually active, with a history of chlamydia and prior dilation and curettage for a medical abortion in 2023. Although she previously had a negative urine pregnancy test at her obstetrician-gynecologist (OBGYN), repeating testing in the emergency department revealed a positive urine pregnancy test and a serum human chorionic gonadotropin (β-hCG) of 1673.14 mIU/mL, with normal laboratory values. Review of systems is positive for abdominal pain and vaginal bleeding, and her physical exam is unremarkable with a soft, nontender, nondistended abdomen. The patient is discharged with resolved symptoms and a positive pregnancy test, with instructions to follow up with her OBGYN. Two days later, the patient returned for repeat testing, demonstrating a β-hCG 1795.46 mIU/mL, with a transvaginal ultrasound recording an ovoid structure adjacent to the right adnexa measuring 2.4 x 2.2 x 2.0 cm. Concern for ectopic pregnancy was raised given the <50% rise in β-hCG over 48 hours, and she underwent laparoscopic right salpingectomy for a confirmed right tubal ectopic pregnancy. Five days later, she returned to the emergency room for evaluation of weakness, dizziness, and increased vaginal bleeding. Repeat evaluation revealed an elevated β-hCG at 2,410.89 mIU/mL. A pelvic ultrasound was performed, which was unremarkable at the time with no evidence of intrauterine pregnancy or adnexal masses. Three days later, the patient received a repeat β-hCG, remarkable at 2,647.88 mIU/mL. A second β-hCG was recorded a day later, along with a follow-up transvaginal ultrasound. The hormone level is reported at 2,568.93, and the ultrasound showed a left adnexal mass separate from the ovary of variable echogenicity with a ring of vascularity, consistent with a left tubal pregnancy. This contralateral tubal pregnancy was successfully treated with methotrexate, and the patient recovered fully. This is a rare clinical presentation that is not well-reported in recent literature. For this patient, the sequential presentation of two tubal pregnancies suggests separate implantation events or delayed fertilization timing, resulting in an atypical bilateral ectopic course requiring different management strategies for each side. This case presents a rare instance of sequential contralateral tubal ectopic pregnancies, initially requiring surgical management on one side and subsequent medical management on the other. The case highlights the importance of maintaining a high index of suspicion for bilateral or sequential tubal pregnancies in at-risk patients, even after treatment of a confirmed unilateral ectopic pregnancy. This report highlights a rare presentation of sequential contralateral tubal ectopic pregnancies and discusses diagnostic and management considerations.

## Introduction

An ectopic pregnancy is one of the most common causes of first-trimester maternal death in developed countries [1]. Diagnosis of these extrauterine pregnancies is complicated due to a wide spectrum of clinical presentation. Patients can be asymptomatic or can be as severe as cases of acute abdomen and hemodynamic shock [1]. Early diagnosis is vital due to the increased risk of maternal morbidity and mortality that occurs due to a ruptured ectopic pregnancy [2]. Over the past two decades, improvements in making early diagnoses have allowed for the definitive medical management of unruptured ectopic pregnancy, possibly even prior to clinical symptoms [2].

The worldwide incidence of ectopic pregnancy is 1-2%, which continues to increase [2]. This is possibly contributed to by pelvic inflammatory disease (PID), ovulation-inducing drugs, previous abdominal-pelvic surgeries, and intrauterine contraceptive devices (IUDs) [2]. All of these listed can increase the risk for an ectopic pregnancy as they can cause damage to the uterus and fallopian tubes and increased ovulation and can impede egg movement. Improved methods in diagnosis and reporting have led to a rise in the incidence of ectopic pregnancy [3].

Bilateral ectopic pregnancy is the rarest type of ectopic pregnancy. It is estimated to be found in 0.000005% of uterine pregnancies and 0.00063-0.00138% of ectopic pregnancies [2]. The first suggested criteria to diagnose a bilateral tubal pregnancy was by Dr. Fishback [4], who declared that there should be a description of fetuses or fetal parts and placental material in both tubes [4]. This has been revised to state that microscopic demonstration of chorionic villi in each tube was sufficient for the diagnosis, as per Dr. Norris [1,4]. This case report will discuss a case of bilateral ectopic pregnancy found in a 26-year-old female, treated as two single sequential ectopic tubal pregnancies. To our knowledge, few reports demonstrate similar clinical courses and treatment methods as presented in this case.

## Case presentation

Our patient is a 26-year-old African American female who presented to the emergency department with right intermittent lower pelvic cramping and vaginal bleeding for the past 13 days. The patient received a Papanicolaou (Pap) smear by her obstetrician-gynecologist (OBGYN) 24 days prior, and the patient reported the bleeding started a few weeks after. The patient is currently sexually active and has a past sexual history of chlamydia, once before 2022 and again recently, treated with antibiotics. The patient also has a surgical history of a dilation and curettage for a medical abortion in August 2023. The patient does not believe she is currently pregnant after a negative urine pregnancy test at the gynecologist. She is gravida 3 para 2 (G3P1112), with her first pregnancy normal with no complications in 2017, and her second pregnancy complicated with chorioamnionitis and induced preterm labor with delivery at 32 weeks in 2022. 

Review of systems is positive for abdominal pain and vaginal bleeding, but negative for nausea, vomiting, lightheadedness, and weakness. Differentials created by the emergency room physician included, but were not limited to, dysfunctional uterine bleeding, ovarian cyst, pelvic inflammatory disease, anemia, and uterine fibroids. Her physical exam is unremarkable with a soft, nontender, nondistended abdomen. 

The emergency department ordered a complete blood count (CBC), basic metabolic panel (BMP), urinalysis (UA), and urine pregnancy test (UPT). The patient has normal lab values with a positive UPT, as well as a serum human chorionic gonadotropin (β-hCG) of 1673.14 mIU/mL. All other working differentials were ruled out because of normal lab values. The patient is discharged with spotting and resolved abdominal pain with a positive UPT, given an obstetrics follow-up, prenatal vitamins, and return precautions discussed. An ultrasound was ordered by the emergency department, but, because of the lack of availability for the scan and resolved clinical symptoms, the patient was discharged without it. 

Two days later, the patient returned to the emergency department to receive a repeat β-hCG with a transvaginal ultrasound. The repeat β-hCG was recorded at 1,795.46 mIU/mL, with the ultrasound report recording an ovoid structure adjacent to the right adnexa measuring 2.4 x 2.2 x 2.0 cm, concerning for an ectopic pregnancy. With the ultrasound results and the less than 50% rise in β-hCG in 48 hours, there is concern for ectopic pregnancy. 

**Figure 1:**
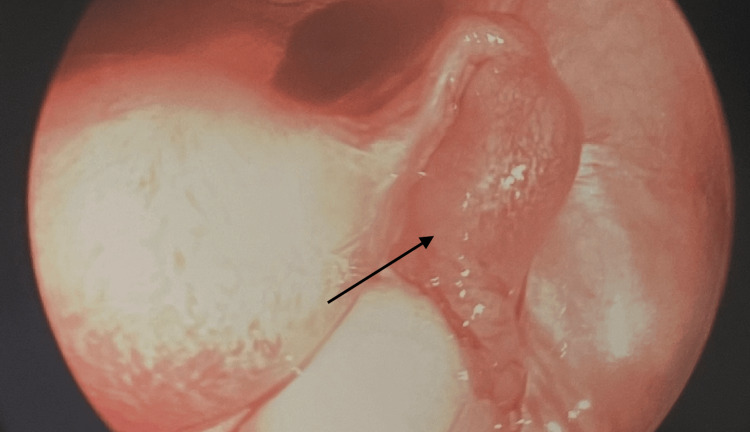
Right fallopian tube containing ectopic pregnancy

The patient was counseled on her options for treatment, and she agreed with the medical team that the best option was a laparoscopic surgery to remove the ectopic pregnancy. The patient was taken to surgery the same day for a laparoscopic right partial salpingectomy. Upon exam, the patient had a normal-appearing uterus and ovaries, as well as a normal-appearing left fallopian tube. The right fallopian tube was grossly enlarged, containing an ectopic pregnancy, which was removed, as seen in Figure 1. The pathology report stated an intraluminal hematoma containing immature degenerating chorionic villi in a pattern consistent with intratubal ectopic pregnancy. The operation went well with no complications, and the patient was discharged home to recover. 

Five days later, the patient returned to the emergency department for evaluation of weakness, dizziness, and increased vaginal bleeding. The patient stated that she has been feeling lightheaded and weak with mild headaches for the last two days. The vaginal bleeding has become heavier with clots. After being examined by the medical team, the patient had a repeat serum pregnancy level, which was reported to have increased to 2,410.89 mIU/mL. A pelvic ultrasound was performed, which was unremarkable at the time with no evidence of intrauterine pregnancy or adnexal masses. The patient's symptoms improved with IV fluids and was set to follow up with the OBGYN physician in 11 days. The emergency department also ordered a repeat β-hCG in two to three days, and the patient was discharged in stable condition. 

**Figure 2:**
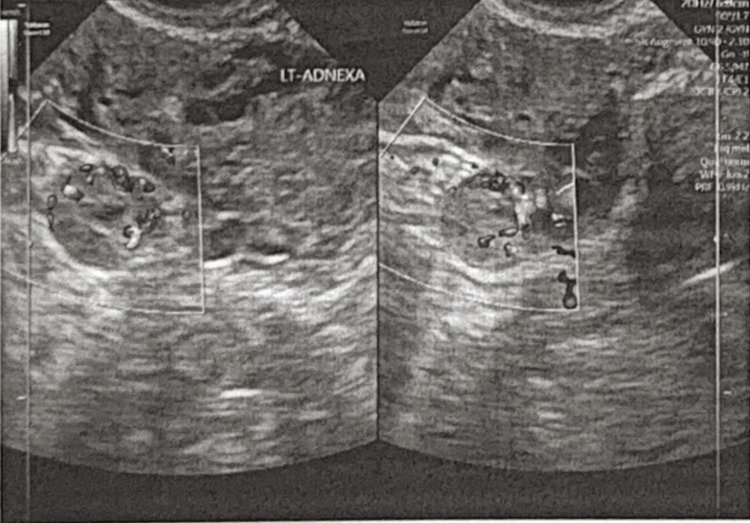
Transvaginal ultrasound showing the left adnexal mass

Three days later, the patient received a repeat β-hCG, remarkable at 2,647.88 mIU/mL. A second β-hCG was recorded a day later, along with a follow-up transabdominal and transvaginal ultrasound. The hormone level was reported at 2,568.93, and the ultrasound showed a left adnexal mass separate from the ovary of variable echogenicity with a ring of vascularity, as seen in Figure 2. 

With this clinical presentation of an increased β-hCG and ultrasound results, the patient was diagnosed with a second ectopic pregnancy of the left fallopian tube. After discussing treatment options, the patient decided to receive a methotrexate injection. She was sent to the emergency department, where she received methotrexate 25 mg/mL chemo injection 91 mg intramuscular (IM), and the patient was set to follow up in the OBGYN clinic. 

Since the patient received a methotrexate injection, she has been monitored to make sure that her β-hCG has returned to normal for a non-pregnant person (<5 mIu/mL). Her “Day 1” value is the baseline before her methotrexate injection, and then she was monitored on days 4 and 7. here should be a 15% fall between day 4 and day 7 to prove that the methotrexate is working correctly. Since that is the case here, with a fall of 36%, the patient continued to get weekly β-hCG levels checked, as seen in the chart presented in Table 1. Over the course of monitoring, the patient's β-hCG levels have dropped to <5 mIU/mL, remarkable for successful treatment with methotrexate. 

**Table 1:** Chart summary of β-hCG levels over time (left ectopic pregnancy)

Time	β-hCG value (mIU/mL)
Day 1 (day of methotrexate injection)	2568.93
Day 4	1717.15
Day 7	1097.35
Day 11	561.83
Day 14	287.98
Day 21	81.92
Day 28	62.89
Day 32	37.70
Day 34	32.99
Day 39	19.16
Day 67	<2

## Discussion

As the rarest form of extrauterine gestation, spontaneous bilateral tubal pregnancy remains a significant diagnostic challenge [2]. Most patients present with clinical features indistinguishable from unilateral ectopic pregnancy, including abdominal pain, vaginal bleeding, and amenorrhea. As a result, diagnosis is frequently delayed or incomplete at initial presentation. They occur spontaneously, as seen in this case, with a very rare case of spontaneous unruptured bilateral tubal pregnancy. Although serial β-hCG measurement and ultrasonography remain cornerstones of ectopic pregnancy diagnosis, these modalities may fail to identify bilateral disease [2]. In many cases, bilateral tubal ectopic pregnancies are not recognized until laparoscopy or laparotomy, underscoring the importance of careful inspection of both fallopian tubes during surgical management [2]. A thorough pelvic examination and knowledge of possible risk factors allow the best course of diagnosis and treatment, especially for preventing tubal rupture [3].

The mechanisms of bilateral tubal ectopic pregnancies have been proposed in various ways, including multiple ovulations, sequential impregnation, and superfetation [4,5,6]. Foster [5] stated that bilateral tubal gestation required multiple ovulations to occur, the oocytes to be fertilized, and the oocytes to implant at sites of tubal damage [5]. Other proposed mechanisms of bilateral tubal pregnancy include transperitoneal migration of trophoblastic tissue from one tube to the other, which is explained in certain cases, such as when Tabachnikoff [7] reported finding fetal tissue in one tube and only villi in the other [5,7]. The latest diagnostic criteria include chorionic villi demonstrated on histopathological examination of tissue obtained from each tube, as presented by Dr. Norris [4].

Treatment of bilateral tubal pregnancy is usually made at the time of surgery with salpingectomy or salpingostomy, and there is a lack of reporting for successful primary medical treatment with methotrexate, as used following a right salpingectomy for the treatment of the left tubal pregnancy in our case [6]. A case from Walter and Buckett [8] reported failed methotrexate treatments, which included bilateral chronic and acute tubal pregnancies that occurred following failed treatment with methotrexate for a previous ectopic pregnancy [5,8]. The more extreme treatment measure includes total abdominal hysterectomy with bilateral salpingo-oophorectomy [5]. Similar to our patient presentation, many cases fail to diagnose bilateral tubal pregnancy during initial surgery [5]. These patients often return with worsening symptoms or symptoms related to rupture of the contralateral tube. This is similar to the patient being discussed in our case, who returned with worsening symptoms five days after initial surgery. 

For those who cannot undergo surgery, want to avoid surgical trauma, or want to offer better fertility prospects, there are some reports that suggest methotrexate treatment [3,9,10]. The use of methotrexate is beneficial in a conservative diagnosis approach; however, there is the risk of missing bilateral tubal pregnancies and potential insufficient treatment [3]. There are approaches to administering methotrexate with local injection during laparoscopic procedures [3,11]. This approach allows a comparative treatment to laparoscopic salpingostomy, as well as eliminating the risk of missing bilaterally under laparoscopic investigation [3,11,12].

It is important to note that in our case, the patient was documented to be sexually active prior to the diagnosis of the right ectopic tubal pregnancy. After the right salpingectomy, the patient was counseled to pause all sexual activities to allow time for proper healing, but it was not explicitly documented whether the patient had intercourse between the diagnosis of the right versus left ectopic tubal pregnancy. As described in the laproscopic salpingectomy, the patient's left fallopian tube was grossly normal-appearing. It is possible that the left tubal pregnancy was present, but was not large enough or far along enough in the clinical course to be seen on gross examination. It is hypothesized that the two tubal pregnancies were fertilized around the same time, with the right shortly before the left. However, without a pathologic analysis of the left fallopian tube, it is hard to determine the timeline of fertilization between the two separate tubal pregnancies. 

In our case, the patient presented with a single ectopic right tubal pregnancy, which was treated with laparoscopic salpingectomy, and a sequential left tubal pregnancy discovered eight to nine days later, which was treated with a methotrexate injection. This treatment regimen does not reflect what is read in most reports of bilateral tubal ectopic pregnancies, but the difference in presentation and potential delay in fertilization of the two separate pregnancies made the case present as sequential tubal pregnancies requiring separate treatment. In other cases reported, such as Andrews and Farrell, success has been noted in the treatment of bilateral tubal pregnancies with conservative tubal surgery [5].

**Figure 3:**
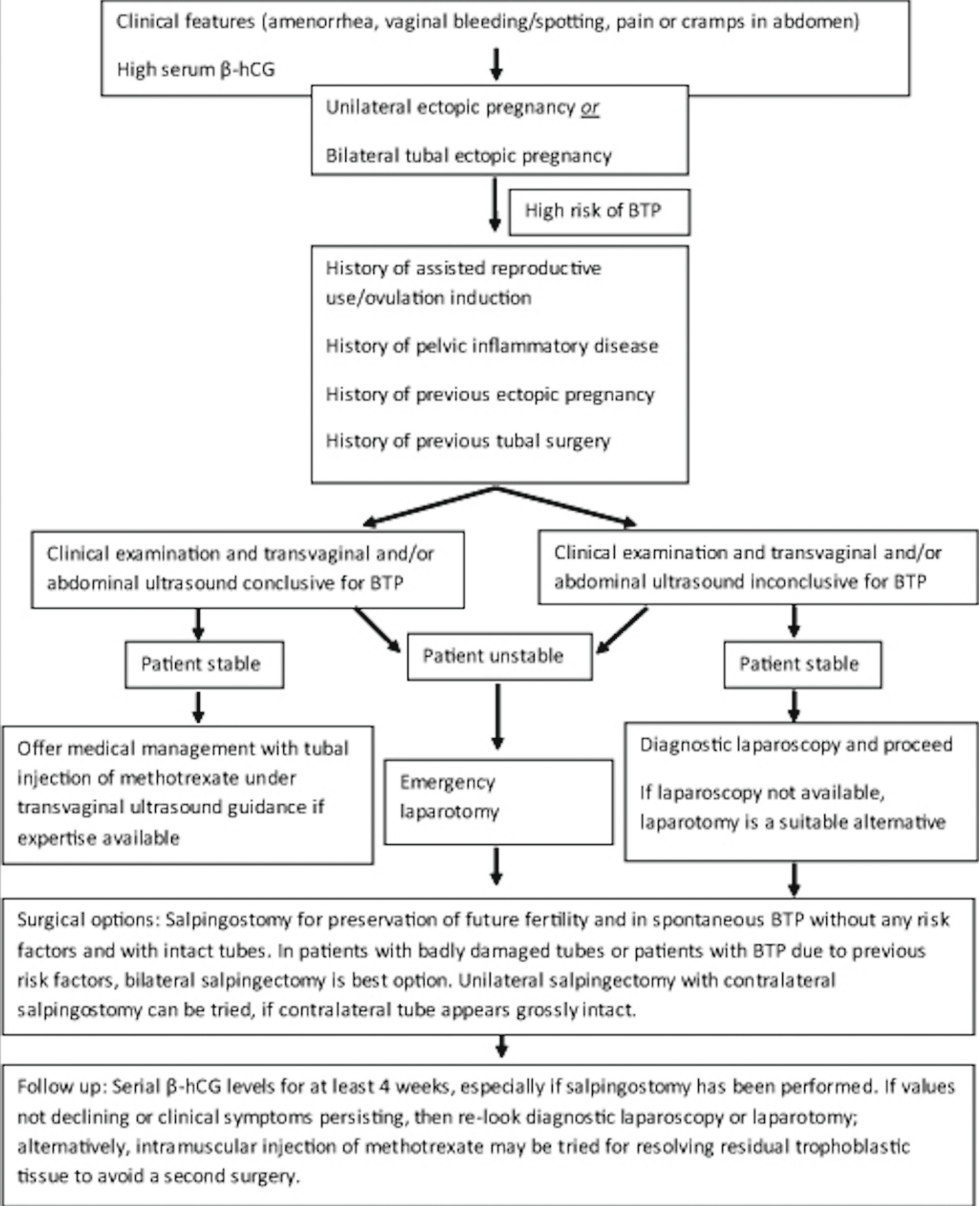
Proposed treatment algorithm for bilateral tubal pregnancies Sourced from Jena et al. [4]

In the absence of any treatment guidelines, a study by Jena et al. proposed a simple management algorithm for women presenting with possible bilateral tubal pregnancies, as seen in Figure 3 [4]. This provides a methodical approach, minimizing the possibility of missing or misdiagnosing bilateral tubal pregnancies [4]. 

## Conclusions

Bilateral tubal ectopic pregnancy is the rarest form of extrauterine pregnancy and is a challenging presentation to diagnose. With its similarity to unilateral ectopic pregnancies, it is easily missed and can result in danger to the patient and their treatment recovery. It is important for clinicians to have a high index of suspicion, as well as thorough inspection of both fallopian tubes during laparoscopy, even if there is a presence of dense adhesions or no evidence of a contralateral ectopic pregnancy, to avoid missing this rare but life-threatening condition. In conclusion, the optimal care strategy for diagnosis and treatment in patients with bilateral tubal pregnancy is laparoscopy, with salpingectomy or salpingostomy. In the case of unilateral tubal pregnancy, it is important to monitor both sides during surgical exploration.
